# The Use of Kaolin as a Prophylactic Treatment to Prevent Columnaris Disease (*Flavobacterium covae*) in Commercial Baitfish and Sportfish Species

**DOI:** 10.3390/vetsci10070441

**Published:** 2023-07-06

**Authors:** Anita M. Kelly, Nilima Renukdas, Louis Matthew Barnett, Benjamin H. Beck, Hisham A. Abdelrahman, Luke A. Roy

**Affiliations:** 1 Alabama Fish Farming Center, School of Fisheries, Aquaculture & Aquatic Sciences, Auburn University, 529 Centreville Street, Greensboro, AL 36744, USA; haa0008@auburn.edu (H.A.A.); royluke@auburn.edu (L.A.R.); 2 UAPB Fish Health Services, University of Arkansas at Pine Bluff, 2001 Hwy 70 East, Lonoke, AR 72086, USA; nilima.renukdas@agriculture.arkansas.gov; 3 Stuttgart National Aquaculture Research Center, USDA-Agricultural Research Service, 2955 Hwy 130 East, Stuttgart, AR 72160, USA; matt.barnett@usda.gov; 4 Aquatic Animal Health Research Unit, USDA-Agricultural Research Service, 990 Wire Road, Auburn, AL 36832, USA; benjamin.beck@usda.gov; 5 Department of Veterinary Hygiene and Management, Faculty of Veterinary Medicine, Cairo University, Giza 12211, Egypt

**Keywords:** kaolin clay, columnaris, *Flavobacterium covae*

## Abstract

**Simple Summary:**

The aquaculture industry suffers large financial losses every year to a bacterial disease known as columnaris. Columnaris can be treated with antibiotics in the feed, but this is expensive and can lead to antibiotic resistance. Alternative inert substances that are inexpensive and that would not lead to antibiotic resistance are needed. One such alternative is kaolin clay. This clay is added to the water and has been shown to bind to columnaris bacteria in laboratory studies. This study used kaolin clay on several species of game and sportfish stocked at commercial rates. When added to the water as a prophylactic treatment, the kaolin clay bound to columnaris. The kaolin clay did not damage the organs of the fish, nor did it cause mortalities. We have shown that kaolin clay is safe to use as a prophylactic treatment in several freshwater fish species raised in Arkansas, USA.

**Abstract:**

Aquaculture farms in Arkansas, USA routinely battle columnaris disease caused by *Flavobacterium covae*. Columnaris is prevalent during stressful events such as feed training and when fish are stocked at high densities in holding vats before sale. Kaolin clay was effective in laboratory trials as a treatment for columnaris in catfish. As a result, fish farmers are interested in applying kaolin products but were hesitant as they feared that the high doses of kaolin clay in vats might negatively affect the gills and overall health of fish. Therefore, we evaluated potential clay concentrations that might be used to prophylactically treat fish in vats. The effects of low to excessively high doses (0, 1, 2, 4, or 8 g/L) of kaolin clay (AkuaPro^TM^, Imerys, GA, USA) were evaluated using a 72 h bioassay conducted in static tanks using *Micropterus salmoides*, *Pomoxis nigromaculatus*, *Lepomis macrochirus*, *Ictalurus punctatus*, *Notemigonus crysoleucas*, and *Pimephales promelas*. Results of these trials revealed a 100% survival rate across all six fish species exposed to kaolin clay at concentrations of up to 8 g/L for 48 h (followed by a 24 h recovery period in clean water) with no adverse effects to eyes, skin, gastrointestinal tract, or liver histology noted at any treatment. In addition, *Micropterus salmoides* analyzed for heavy metals due to exposure to the clay indicated that concentrations did not differ from control fish.

## 1. Introduction

Columnaris disease affects many wild and cultured fish species worldwide [1,2,3,4,5]. In the United States, columnaris disease causes millions of dollars in losses in the aquaculture industry [2,6]. Columnaris disease is caused by a Gram-negative, yellow-pigmented bacterium (*Flavobacterium covae*; formerly *F. columnare* genomovar II [7]). This bacterium is an opportunistic pathogen, ubiquitous in freshwater environments, and may survive for prolonged periods in water [8]. Columnaris disease outbreaks can occur without any stressor [9] or because of environmental stressors, such as crowding, high stocking densities, and temperature stress [10,11,12,13].

Despite the large monetary losses due to columnaris disease, there are limited treatments available. Antimicrobial drugs have shown some ability to combat columnaris disease, but currently, their use in aquaculture is highly scrutinized. In some cases, the use of antibiotics prophylactically occurs with little to no regulation. This inappropriate use of antibiotics is performed to prevent or mitigate bacterial infections resulting from sanitary shortcomings in fish rearing [14]. The extensive use of antibiotics in aquaculture has resulted in drug resistance by some aquaculture pathogens [15]. Moreover, the potential impacts on human health from drug-resistant bacteria and the transfer of resistance to human-associated bacteria is a major concern [16]. Indeed, antibiotic resistance to several clinically important antibiotics such as quinolones and tetracyclines has been documented in *Flavobacterium* spp. isolates originating from ornamental fish [16]. Clearly, alternative mitigating strategies are desperately needed for columnaris disease and other bacterial pathogens affecting cultured fish.

Clay minerals are abundant in nature and their absorptive capabilities have been exploited in various cosmetics and pharmaceutical formulations [17,18,19,20,21]. The advantages of clay minerals include their natural abundance, low cost, and environmental friendliness. Clay minerals have been used as excellent adsorbents for bacteria removal in water, which has practical applications in wastewater treatment [22] and environmental bioremediation [23]. The microorganism quantity adsorbed onto kaolinite clay minerals decreases with an increase in the pH of the solution due to an increase in the repulsive force between the clay mineral and the bacteria [24]. Under neutral pH conditions, the surface charge on the clay mineral, including the non-electrostatic forces such as hydrogen bonds, Van der Waals force, and hydrophobic interactions, are responsible for the antimicrobial activity [24,25].

Kaolinite clay was found to adsorb *Pseudomonas putida*, and the adsorption capacity increased with increasing temperature from 15 to 35 °C [24]. Several factors impact bacteria adhesion onto adsorbent clay surfaces or their flocculation, including the type of bacteria and properties of the adsorbent clay surface (surface chemistry, surface charge, and composition), and environmental properties such as proteins, roughness, and bacterial hydrophobicity [26,27,28].

*Flavobacterium covae* is unique because it is primarily an external bacterium affecting the external mucosal surfaces of fish [29,30]. Consequently, the external nature of this bacteria makes it a prime candidate for surface-acting compounds, such as kaolin clay. Kaolin is an inert substance with a history of medicinal use in humans, mainly as an adsorbent of pathogenic bacteria, particularly gastrointestinal diseases [31,32]. The use of kaolin in aquaculture is not new. It has been used as a bulking agent in pelleted feeds [33,34] and to reduce adhesiveness and clumping of eggs in hatchery operations [35]. Additionally, many *Flavobacterium* spp. are highly susceptible to adsorption by kaolin [36,37].

Kaolin has been used on an experimental basis as a prophylactic and for treating *Flavobacterium covae* in Channel Catfish *Ictalurus punctatus*. Beck et al. [38] reported higher survival (*p* < 0.001) in Channel Catfish treated with 1 g/L kaolin compared to a control following an experimental challenge. In the same study, the incubation of kaolin with *F. covae* using in vitro techniques resulted in fewer *F. covae* cells in culture supernatants. They concluded that kaolin could reduce gill pathologies and bacterial attachment to key tissues and increase survival in Channel Catfish exposed to *F. covae*. Kaolin has also been proven to be effective in blocking chemotaxis and adherence of *Aeromonas hydrophila* in the mucus of Channel Catfish [39]. Kaolin treatment at a rate of 0.1% increased survival (*p* < 0.05) compared to control (untreated) Channel Catfish in the same study [39].

After hearing of the positive benefits of kaolin for the catfish industry, sportfish and baitfish farmers in Arkansas, USA expressed interest in the potential of kaolin to reduce the incidence of columnaris on their farms, particularly in vats designed to hold fish before sale. Following the harvest of commercial ponds, farmers raising Fathead Minnows (*Pimephales promelas*), Golden Shiners (*Notemigonus crysoleucas*), Goldfish (*Carassius auratus*), Channel Catfish, and centrarchids, including Largemouth Bass (*Micropterus salmoides*), Bluegill (*Lepomis macrochirus*), Redear Sunfish (*Lepomis microlophus*), Black Crappie (*Pomoxis nigromaculatus*), and other species are transported in hauling tanks to holding sheds on the farm to warehouse fish in vats from several days to a few weeks depending on the time of year and market demand before sale. During this holding period, columnaris infections are common, resulting in mortality and significant financial losses for the commercial producer. Based on the initial success of kaolin clay in preventing columnaris disease in catfish, a small number of Arkansas farmers have experimented with comparable dose treatments (~1 g/L kaolin clay for a 1 h immersion treatment) on many different species in their vat holding systems. Typically, water flow to vats is ceased for one hour to administer the kaolin clay treatment, after which the water is turned back on, and the kaolin clay is flushed out of the vat over time. Anecdotal evidence from commercial producers and preliminary data (Kelly, unpublished data) have suggested that a 1 g/L treatment effectively reduces the incidence of columnaris in fish held in commercial vats. However, treatment at the rates [38] recommended for Channel Catfish can make the water in the holding vats cloudy. Farmers have expressed concern about potential adverse effects on the gills of fish undergoing a 1 g/L kaolin immersion treatment.

To address this question posed by commercial producers, a short-term 72 h bioassay study was designed to assess the impact of standard and much higher treatment rates of kaolin clay on the survival and eye, skin, gill, gastrointestinal tract, and liver histology of commonly raised baitfish and sportfish species, including Fathead Minnow, Golden Shiner, Largemouth Bass, Black Crappie, Bluegill, and Channel Catfish, following exposure to culture water containing various aqueous concentrations of kaolin clay.

## 2. Materials and Methods

### 2.1. Experimental Design

Six separate 72 h bioassays were conducted with Channel Catfish, Largemouth Bass, Black Crappie, Bluegill, Fathead Minnows, and Golden Shiners. Each trial was performed using the same methodology. These experiments were carried out at the Lonoke Agricultural Center in Lonoke, Arkansas. Fish were stocked into 20 static 37-L aquaria with culture water previously prepared with de-chlorinated water and different kaolin concentrations (0, 1, 2, 4, or 8 g/L; Table 1). Kaolin clay (AkuaPro^TM^, Imerys, GA, USA) was added to tanks at least 24 h before starting each trial to allow for adequate mixing. Twenty-five fish were stocked per aquarium in the Channel Catfish, Black Crappie, Bluegill, Fathead Minnow, and Golden Shiner bioassays, while twelve fish were stocked per aquarium in the Largemouth Bass bioassay. These bioassays were run separately by species. Fish were stocked at commercial production densities in g/L as follows: Channel Catfish 2.4, Black Crappie 4.5, Bluegill 9.9, Fathead Minnow 0.7, Golden Shiner 1.7, and Largemouth Bass 1.8. Each treatment had four replicates, with the 0 g/L treatment serving as the control. The number of fish used per species in each bioassay depended on the availability of fish from local hatcheries. At the beginning of each bioassay, fish (*n* = 12–25) from each species were sampled to determine individual length and weight, which are shown in Table 1.

Each aquarium was equipped with air stones supplied with aeration from a regenerative blower. Following exposure of fish to different kaolin treatments for 48 h, they were pooled by treatment (75 fish each for Channel Catfish, Black Crappie, Bluegill, Fathead Minnow, and Golden Shiner; and 36 Largemouth Bass) and moved to larger (189 L) separate clean water tanks for an additional 24 h to assess recovery. Throughout the experiment, dissolved oxygen and temperature were monitored daily, whereas total alkalinity and total hardness were assessed in each tank before and after the trial. The fish were not fed throughout the 72 h experiment.

These 72 h bioassays were designed to evaluate a 48 h immersion treatment using different concentrations of kaolin clay followed by a 24 h recovery period (72 total hours). The experiment was designed this way to answer whether an immersion treatment of kaolin could adversely affect the survival or eye, skin, gill, gastrointestinal, and liver health of commercially raised fish. In addition, Largemouth Bass exposed to the various kaolin concentrations in this study were analyzed for metals to determine if kaolin increased metal concentrations in fish. Hence, the bioassays were designed to calm the fears of farmers and prove that a 1 g/L vat treatment of kaolin would not be harmful in any way to fish.

### 2.2. Histology

Either whole fish or tissues from three fish from each replicate tank per treatment were collected and fixed into 10% neutral buffered formalin. After 24–48 h of fixation, gills were briefly rinsed with water, transferred to 70% isopropanol and stored in routine paraffin embedding with a Leica TP1020 tissue processor (Leica Biosystems, Deer Park, IL, USA). Whole fish or tissues from each treatment were embedded in paraffin and sectioned with a Leica RM2135 microtome to 5–6 µm, mounted on slides and stained with hematoxylin and eosin. Eyes, skin, gill, gastrointestinal tract, and liver tissues were then examined using Aperio ImageScope ver 12.4.6 (Leica Biosystems, Vista, CA, USA) for the effect of kaolin clay on these structures.

### 2.3. Electron Microscopy of Channel Catfish Skin

Skin samples were collected only from three channel catfish in each treatment tank to determine if kaolin samples stuck to the bacteria and the skin. The skin samples were cut into small pieces and fixed in 2.5–3% glutaraldehyde prepared in 0.1 M sodium cacodylate buffer (pH 7.2) for 4 h at 40 °C. The tissues were then washed in 0.1 M sodium cacodylate buffer for 15–30 min, dehydrated in ascending grades of acetone with two changes of 15 min each and dried. The dried samples were secured horizontally to brass stubs with double adhesive tape. Samples were placed on mica and sputter coated with platinum. Images were obtained with a field emission scanning electron microscope (JEOL 6700F, JOEL Ltd., Tokyo, Japan) at an accelerating voltage of 15 kV.

### 2.4. Whole Body Metal Analysis of Largemouth Bass

For metal analysis, three Largemouth Bass individuals from each replicate tank were euthanized in MS-222, and whole bodies were dried at 150 °C in a convection oven for 24 h. The three fish from the same tank were then ground and thoroughly mixed. One hundred grams of the sample mixture was placed into an amber vial and sent to Alpha Analytical (Mansfield, MA, USA) for whole-body analysis of arsenic, chromium, copper, mercury, lead, and zinc. Largemouth Bass were the only species analyzed for heavy metals due to monetary constraints.

### 2.5. Statistical Analysis

To test the effect of kaolin treatments on the concentration of each metal, a one-way analysis of variance test (ANOVA) was used. The Shapiro–Wilk test was utilized for the normality analysis of the variables and Levene’s test was used to evaluate the homogeneity of variances (HOV; homoscedasticity). When normality and/or HOV assumptions were violated, the variance in the heavy metal concentration among kaolin treatments was analyzed using a non-parametric test (Kruskal–Wallis test). If there were significant differences among treatments, post hoc pairwise comparisons were performed using Tukey’s studentized range—HSD (or Dwass–Steel–Critchlow–Fligner (DSCF) for Kruskal–Wallis). Statistical significance was set at *p* < 0.05. All data were presented as the mean ± standard error of the mean (*SE*). All analyses were performed with SAS^®^ version 9.4 [40]. All graphs were plotted in SigmaPlot^®^ software (version 14.5; Systat Software Inc., San Jose, CA, USA).

## 3. Results

Following the 72 h bioassays with Largemouth Bass, Black Crappie, Channel Catfish, Bluegill, Fathead Minnows, and Golden Shiners, 100% survival occurred in all treatment groups (Table 1).

Throughout the trial, water quality remained within acceptable limits for the culture of these species (Table 2). Total hardness and alkalinity for all trials were (mean ± SE) 239.0 ± 7.6 and 123.2 ± 3.42. No significant differences occurred between any of the treatments or before or after the trial.

**Figure 1:**
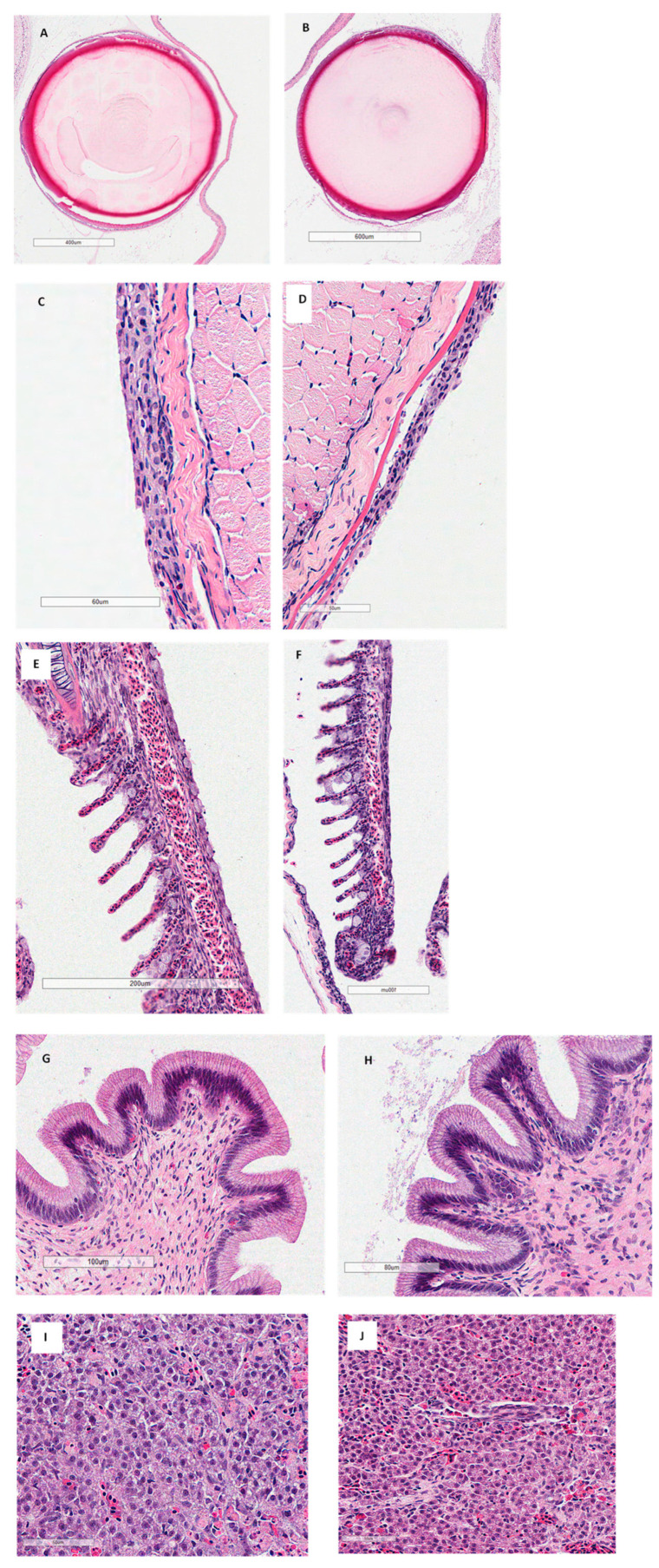
Histology of eyes (**A**), skin (**C**), gills (**E**), gastrointestinal tract (**G**), and liver (**I**) of Largemouth Bass *Micropterus salmoides* controls (not exposed to kaolin clay). Fish exposed to the highest concentrations of kaolin clay (8 g/L) are shown in (**B**) eyes, (**D**) skin, (**F**) gills, (**H**) gastrointestinal tract, and (**J**) liver. Eyes were observed under 40X, skin 40X, gills 20X, gastrointestinal tract 20X, and liver 40X. No abnormal histological findings attributed to kaolin were found.

Histological examination of the gills in all the fish species tested showed that kaolin-treated fish did not have significant pathological findings on the eyes, skin, gills, or gastrointestinal tract regardless of treatment (Figure 1; Largemouth Bass only; Table 3). Abnormalities in the histology of the organs were attributed to being artifacts of the slide-making process by the pathologist.

**Figure 2:**
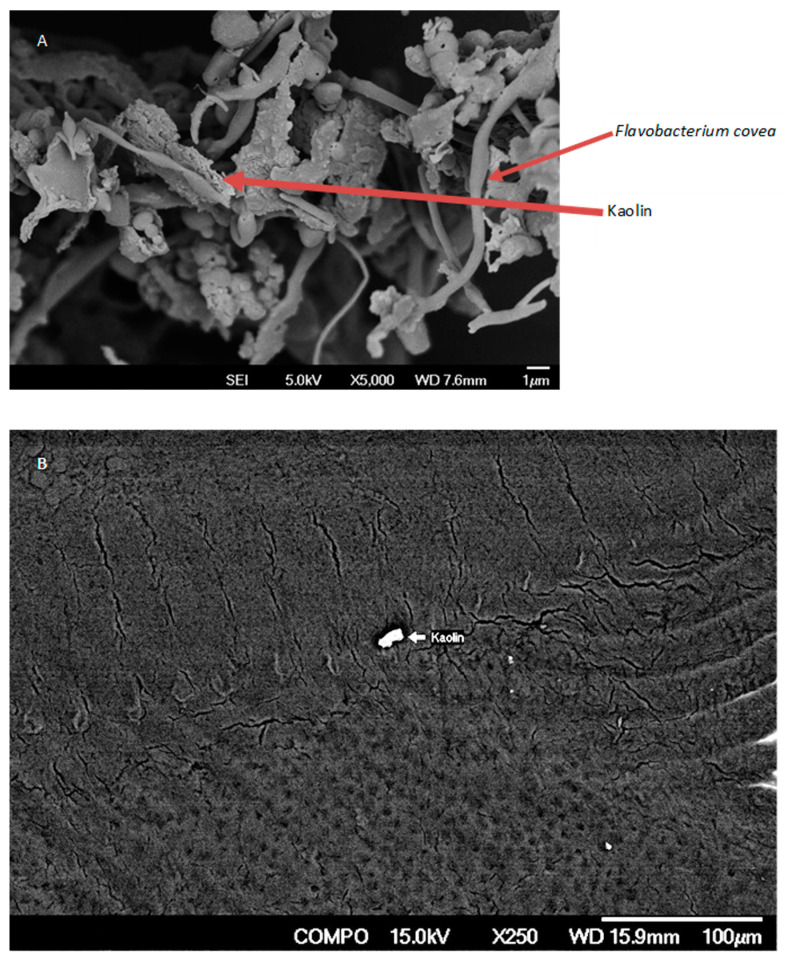
Scanning electron micrograph of kaolin adhering to *Flavobacterium covae* (**A**), but not to the skin of fish (**B**).

The scanning electron microscopy revealed that the kaolin does stick to Flavobacterium covae but not to the skin of channel catfish (Figure 2).

**Figure 3:**
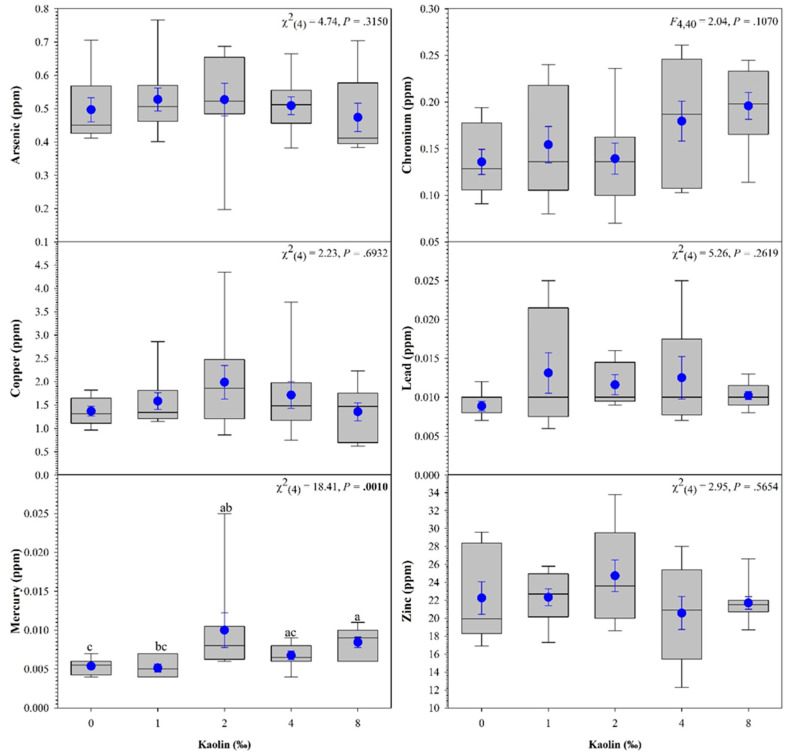
Effects of different kaolin treatments on whole body concentration of heavy metals in Largemouth Bass *Micropterus salmoides*. Within each box plot, horizontal line indicates median, symbol indicates the mean, and error bars around the symbol represent standard error of the mean. Within each figure, data with different lowercase letters are significantly different among treatments at *p* < 0.05.

Metal analyses of Largemouth Bass revealed a significant difference in mercury concentration (Figure 3). The 8 g/L treatment group had significantly higher concentrations of mercury compared to the control and 1 g/L groups.

## 4. Discussion

Using kaolin clay as a prophylactic treatment exposes fish temporarily to high concentrations of clay (suspended solids) in the vat or tank in which they are treated. This study examined the exposure of various commercially raised species to high concentrations of clay for a short time (48 h) and examined recovery following an additional 24 h. Commercial aquaculture producers in Arkansas have experimented with kaolin treatments (1 g/L) for a maximum of 1 h in vats to prevent columnaris, following commercial harvest and during holding periods prior to sale. Hence, this study examined a worst-case scenario regarding exposure time and concentration to ascertain the potential adverse effects of short-term kaolin clay exposure. Overall, the kaolin clay concentrations evaluated in this study had no measurable adverse effects on any of the six species under the examined experimental conditions.

Several authors have examined the impact of total suspended solids in fish, particularly in natural systems that have been impaired by anthropogenic inputs [41]. In general, aqueous concentrations of suspended solids must be high to have a direct negative effect on fish [42,43,44]. Wallen [42] performed a series of experiments on 16 species using montmorillonite clay concentrations at turbidities ranging from 20,000–225,000 ppm. Total suspended solids have been determined to cause gill damage, increase susceptibility to predation, affect swimming performance, reduce feeding, reduce growth, and increase cortisol concentrations in some species of fish [41,43,44,45,46,47,48,49,50,51,52]. The negative effects of total suspended solids on fish are a product of exposure duration and concentration. Surprisingly, many fish species are quite resilient in their tolerance to high concentrations of total suspended solids for short and even more extended exposure durations.

The gill is considered the most sensitive organ in fish when exposed to suspended solids. Herbert and Merkens [43] examined the gills of rainbow trout exposed to 270 ppm China clay and 810 ppm diatomaceous earth for several months. In some cases, the cells of the respiratory epithelium of some fish examined in both treatments were noted to be much thicker than typical gills. Additionally, adjacent lamellae were fused, often at the tips. However, other fish exposed throughout the trial showed no effects, even after nine months. Goldes et al. [53] reported that the exposure of juvenile rainbow trout to different concentrations of suspended clay (36, 171, 1017, and 4887 mg/L) had little direct effect on gill structure following histological examination. Our study had similar results, as none of the treatment concentrations showed negative effects.

The use of kaolin clay as a prophylactic treatment at 1 g/L for a short time (acute exposure) is a very different situation than fish residing or being cultured in water with chronically high total suspended solids, which can affect growth, survival, predation, feeding, reproduction, and other factors long-term. Most studies documenting the deleterious effects of total suspended solids on fish have focused on long-term exposure, often at high concentrations of total suspended sediments. Newcombe and MacDonald [47] highlighted the importance of designing studies that evaluate the duration of exposure to high concentrations of suspended solids to distinguish potential effects. Often, fish can survive exposure to high concentrations of suspended solids for extended periods, albeit sublethal physiological stress and increased susceptibility to bacterial pathogens have been reported in some species, including salmonids [46].

The lack of mortality in this study was not unexpected. Similar results were obtained in a study conducted evaluating the exposure of eleven estuarine fishes to kaolin clay [54]. In that study, all fish survived when exposed to concentrations of kaolinite as high as 140 g/L. It is worth noting that modified kaolin clays have been used to treat harmful algal blooms in oceans since the late 1990s [55]. Consequently, research has demonstrated that clay particles used in these ecosystems are essentially nonharmful to fish species, including embryos [56].

We presented only the Largemouth Bass histology for this study, as it was representative of the results obtained for the other fish species. The pathology reports did not highlight any significant differences between the controls and any of the kaolin concentrations used in this study. Therefore, we only presented the histology pictures of the control and the highest concentration used, which was 8 g/L.

A comparison of the effects of the kaolin clay between species of baitfish and sportfish was not conducted in this study. Since no mortalities occurred in any of the test species in this study, neither an LD_50_ nor a no observable effect concentration (NOEC) could be obtained. The fish used in this study had similar weights within a species making the bioassay on that species relevant. Although we used different sizes and stocking densities, the objective was to determine the effect of kaolin clay on several commercially produced species at concentrations much higher than the 1 g/L concentration currently used by producers and at sizes that would be held for sale. Since these immersion treatments were much longer and at higher concentrations compared to what commercial fish farms are using, it allowed for an assessment of fish health under a much more extreme time frame and at higher concentrations than would be used by a commercial farmer.

The U.S. Food and Drug Administration (FDA) has recently acknowledged that arsenic, lead, mercury, and cadmium are human health hazards. However, since these heavy metals occur naturally in the environment, the FDA is monitoring these compounds in foods and developing guidance for their reduction. To date, only mercury concentration in food has a guidance document.

The FDA has established methyl mercury, expressed as mercury, concentrations to not exceed 1 ppm in the edible portion [57]. All the whole Largemouth Bass sampled in this study had concentrations well below the regulatory limits. Although the mercury concentrations were significantly higher in the fish treated with 2 g/L of kaolin, the increase is likely not due to the kaolin treatment. Largemouth Bass are carnivorous predators, with cannibalism beginning nine days after swim-up [58]. Two fish in the 2 g/L kaolin treatment group had higher concentrations of mercury (0.025 and 0.011) than the other fish within the group. We speculate that these fish may have been cannibalistic during the early life stages prior to feed training. Since mercury accumulates in the food chain, these fish could have potentially accumulated mercury. However, more research is needed to confirm this hypothesis.

## 5. Conclusions

Results of this study support using kaolin as a prophylactic treatment in baitfish and sportfish stocked in vats prior to sale to prevent columnaris at the concentration (1 g/L) and exposure duration (1 h) currently used by commercial aquaculture producers. No adverse effects were observed in any of the species examined in this study at concentrations much higher (up to 8 g/L) and for a much longer exposure duration (48 h) than what is used by commercial aquaculture producers.

## Figures and Tables

**Table 1:** Total body length (cm), body weight (g), and survival (%) of Channel Catfish *Ictalurus punctatus*, Black Crappie *Pomoxis nigromaculatus*, Largemouth Bass *Micropterus salmoides*, Bluegill *Lepomis macrochirus*, Golden Shiners *Notemigonus crysoleucas*, and Fathead Minnows *Pimephales promelas* subjected to a 72 h bioassay with different concentrations (0, 1, 2, 4, and 8 g/L) of kaolin clay. No mortality was observed across any of the experimental treatments in any of the fish species examined. Values represent the mean ± standard error.

Fish Species	Average Length (cm)	Average Weight (g)	Survival (%)
Channel Catfish	7.9 ± 0.75	3.5 ± 1.20	100
Black Crappie	2.7 ± 0.78	6.6 ± 0.59	100
Largemouth Bass	8.2 ± 0.69	5.6 ± 1.41	100
Bluegill	9.4 ± 0.72	14.6 ± 3.24	100
Golden Shiner	6.8 ± 0.68	2.5 ± 0.91	100
Fathead Minnow	4.7 ± 0.27	1.0 ± 0.16	100

**Table 2:** Dissolved oxygen (mg/L) and temperature (°C) in 72 h bioassays with Channel Catfish *Ictalurus punctatus*, Black Crappie *Pomoxis nigromaculatus*, Largemouth Bass *Micropterus salmoides*, Bluegill *Lepomis macrochirus*, Golden Shiners *Notemigonus crysoleucas*, and Fathead Minnows *Pimephales promelas* stocked in aquaria with different concentrations (0, 1, 2, 4, and 8 g/L) of kaolin clay. Values represent the mean ± standard error.

Bioassay	Dissolved Oxygen (%)	Temperature (°C)
Channel Catfish	125 ± 3.71	17.8 ± 0.27
Black Crappie	125 ± 6.94	18.1 ± 0.93
Largemouth Bass	130 ± 5.34	16.2 ± 1.50
Bluegill	124 ± 4.97	18.7 ± 0.29
Golden Shiner	122 ± 1.17	17.8 ± 0.20
Fathead Minnow	122 ± 3.18	17.8 ± 0.25

**Table 3:** Histopathological findings in eyes, skin, gills, and gastrointestinal tract of ten fish each for Channel Catfish *Ictalurus punctatus*, Black Crappie *Pomoxis nigromaculatus*, Bluegill *Lepomis macrochirus*, Golden Shiners *Notemigonus crysoleucas*, and Fathead Minnows *Pimephales promelas* stocked in aquaria with different concentrations (0, 1, 2, 4, and 8 g/L) of kaolin clay. Only the control (0 g/L) and the 8 g/L results are presented for simplicity.

Species	Control (0 g/L Kaolin)	8 g/L Kaolin
Channel Catfish	Gills: Irregular epithelial surface and focal loss of outer epithelium; Skin: Focal thinning or loss of the epidermis was present in the flank region (suspected sampling artifact); Eyes and gastrointestinal system: No significant findings.	Gills: Irregular epithelial surface and focal loss of outer epithelium; Skin: Focal thinning or loss of the epidermis was present in the flank region (suspected sampling artifact); Eyes, gastrointestinal system: No significant findings.
Black Crappie	Gills: Irregular epithelial surface and focal loss of outer epithelium; Skin: Focal thinning or loss of the epidermis was present in the flank region (possibly related to sampling artifact); Eyes, gastrointestinal system: No significant findings.	Gills: Irregular epithelial surface and focal loss of outer epithelium; Skin: Some irregular epidermal cells in the outer epidermis; Brain, kidney, spleen, heart, gastrointestinal system: No significant findings.
Bluegill	Gills: Irregular epithelial surface and focal loss of outer epithelium; Skin: Focal loss of the outer epidermis at the flank region; Eyes, gastrointestinal system: No significant findings.	Skin: Focal thinning or loss of the epidermis was present in the flank region (possibly related to sampling artifact); Eyes, gastrointestinal system: No significant findings.
Golden Shiner	Gill, skin, intestine, eye: No significant findings.	Gill, skin, eye: No significant findings; Gastrointestinal tract: Small protozoan parasites associated with gut epithelial surface.
Fathead Minnow	Gill, skin, gastrointestinal tract intestine, eye: No significant findings.	Gill, liver, stomach, pancreas, intestine, eye: No significant findings.

## Data Availability

Data are available upon reasonable request from the authors.
